# From Zombies to Smart Devices: The Evolution of Dye-Sensitized Solar Cells for IoT Applications

**DOI:** 10.1021/acsaem.5c00624

**Published:** 2025-07-15

**Authors:** Kezia Sasitharan, Marina Freitag

**Affiliations:** School of Natural and Environmental Science, 5994 Newcastle University, Newcastle upon Tyne NE1 7RU, United Kingdom

**Keywords:** Dye-sensitized solar cells, Internet of Things, Copper complexes, Indoor photovoltaics, Hole transport materials, Quasi-solid electrolytes

## Abstract

The evolution of dye-sensitized solar cells (DSCs) has been fundamentally shaped by advances in charge transport materials, with copper-based coordination complexes enabling efficient redox mediation and, uniquely, the in situ formation of solid-state hole transport networks. This Spotlight traces the materials design principles underpinning the “zombie” DSC, devices that maintain or even improve performance after the spontaneous solidification of a liquid electrolyte within the mesoporous TiO_2_ scaffold. Building on the 2015 demonstration of copper–phenanthroline complexes forming self-assembled, conductive matrices, we discuss the interplay of ligand rigidity, redox potential, and reorganization energy and compare with recent breakthroughs in cobalt and iron polypyridyl complexes as well as polyiodide systems. Advances in ligand engineering have yielded amorphous, robust hole conductors with conductivities exceeding 1 mS cm^–1^ and power conversion efficiencies up to 38% under 1000 lx indoor light, with less than 5% efficiency loss after 1000 h continuous operation. Rapid, scalable processing, such as direct electrode drying and microwave-assisted evaporation, now enables large-area modules to be fabricated in under an hour, with stable integration into Internet of Things (IoT) sensor systems. By uniting molecular design, process optimization, and real-world device integration, zombie DSCs offer a compelling route to sustainable, high-performance indoor photovoltaics and self-powered electronics. Envisioning a new phase of IoT, these DSCs can power small, autonomously operating sensor modules. Moreover, integrating local intelligence, such as resource-limited neural networks, allows on-device analytics and real-time energy management, boosting efficiency while relying solely on ambient light.

## Graphical abstract

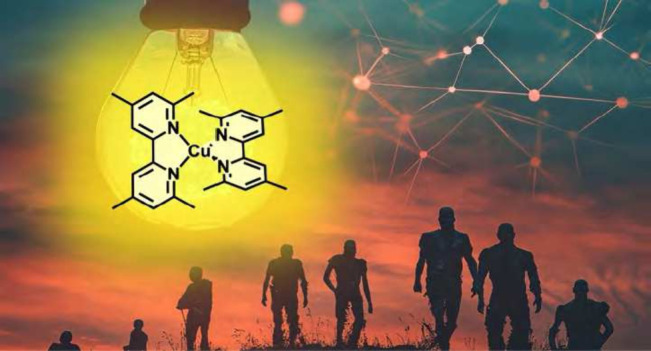

## Introduction

The rapid expansion of the Internet of Things (IoT) presents opportunities for implementing sustainable power solutions that can complement existing power infrastructure. While IoT devices currently operate using various power sources, including batteries and grid connections, autonomous energy harvesting solutions offer advantages for specific applications, particularly in remote or difficult-to-access locations where traditional power sources face practical limitations.^1^ With an exponentially growing number of IoT sensors envisioned in smart homes and industries, conventional push button batteries pose severe logistical, cost, and environmental concerns.^2^

A majority of future IoT devices will operate indoors, under artificial lighting conditions, where illuminance levels typically range from 200 to 1000 lx, a small fraction (<1%) of full sun (AM1.5G, 100 mW cm^–2^).^3^ Conventional semiconductor solar cells (e.g., silicon, GaAs) are less efficient in this regime due to spectral mismatch and insufficient photocurrent generation.^4^ In contrast, organic photovoltaics (OPV),^5–7^ perovskites,^8,9^ and especially dye-sensitized solar cells (DSCs) have gained prominence for indoor applications, owing to their ability to absorb and convert diffuse visible light, with tailor-made dyes spanning the 400–650 nm range.^10^

The fundamental principles of dye sensitization in photoelectrochemical cells were established in the 1960s and 1970s, with O’Regan and Grätzel’s 1991 work introducing mesoporous TiO_2_ thin films as a pivotal advancement, enabling enhanced light harvesting through increased surface area.^11,12^ DSCs operate via a nanocrystalline TiO_2_ scaffold sensitized with molecular dyes: photoexcited electrons are injected into TiO_2_ and the oxidized dye is regenerated by a redox mediator in the electrolyte or hole-transport material (HTM).^11^ Early DSCs predominantly utilized the iodide/triiodide redox couple, which has become a cornerstone of the field, demonstrating remarkable long-term stability through recent innovations. For example, Kato et al. demonstrated that DSC modules can retain 75% of their initial performance after 12 years of outdoor operation,^13^ while Godfroy et al. reported only 20% performance loss after 7000 h in standardized ISOS-L2 testing.^14^ These achievements, particularly through the use of ionic liquid electrolytes, have established I^–^/I_3_^–^-based DSCs as highly stable photovoltaic systems. Although the moderate open-circuit voltage (*V*_OC_ < 0.8 V) of these systems presents limitations for some applications, this trade-off is often justified by their superior long-term stability compared to alternatives.^12^

To overcome voltage limitations and further improve performance, the field progressed to transition-metal-based redox mediators. In 2005, Fukuzumi’s group introduced copper-based phenanthroline complexes ([Cu­(dmp)_2_]^2+/+^) with faster electron transfer and reduced mediator absorption.^15^ Over the following decade, extensive work by Freitag and colleagues advanced copper bipyridine and phenanthroline derivatives, pushing redox potentials closer to 1.0 V while maintaining minimal reorganization energies.^16^ The archetypal [Cu­(tmby)_2_]^2+/+^ (tmby = 4,4′,6,6′-tetramethyl-2,2′-bipyridine) can achieve record 13–15% PCEs under full sun,^17–23^ and has truly revolutionized device performance under low-light conditions: copper complexes offer higher redox potentials (*V*_OC_ ∼ 1.0 V), fast dye regeneration with a driving force as low as 0.1 eV, and tunability to match both the dye’s energetics and the semiconductor conduction band.^24–26^

A transformative milestone was the discovery of “zombie” DSCs.^27^ In these devices, the Cu­(II/I) electrolyte, initially in liquid form, reorganizes into a quasi-solid or fully solid HTM after a controlled evaporation process, eliminating leakage and simplifying encapsulation.^28,29^ This self-assembled solid-state HTM allows fabrication of thicker TiO_2_ layers (>1 μm), enhancing light harvesting and yielding exceptional indoor PCE values (up to 38% at 1000 lx).^30^ Given the narrow emission bands of indoor light sources (primary peaks at 456 and 579 nm), the theoretical maximum PCE under these conditions is 52.3%, calculated using detailed balance analysis accounting for photon recycling effects. Under monochromatic illumination (530 nm, 0.1 mW cm^–2^), devices demonstrate external quantum efficiencies of 91%, approaching the theoretical limit for single-junction devices. Notably, Soman and colleagues demonstrated power conversion efficiencies of 35.6% under 1000 lx warm white illumination using asymmetric dual species Cu­(II)/Cu­(I) electrolytes.^31^

The emergence of copper-based “zombie” DSCs predated and inspired a wave of new solid-state and quasi-solid-state mediators. Bach and co-workers subsequently introduced cobalt polypyridyl complexes, achieving up to 5.7% efficiency in DSCs, and later developed iron hexadentate complexes as transparent, solution-processable solid-state HTMs for perovskite solar cells, reporting conductivities up to 0.24 mS m^–1^ and PCEs of 2.2%.^32,33^ In parallel, Robertson and colleagues developed rapid-zombie polyiodide DSCs, which can be fabricated by direct ambient-air drying before cell assembly, achieving PCEs of 5% and unprecedented long-term stability, with some devices showing increased efficiency after 12 months of dark storage.^34^

Advances in ligand engineering, process optimization, and the mechanistic understanding of solidification have yielded amorphous, robust hole conductors with conductivities exceeding 1 mS cm^–1^ and high PCEs, with less than 5% loss after 1,000 h continuous operation.^35^ Rapid, scalable processing, such as direct electrode drying and microwave-assisted evaporation, now enables large-area modules to be fabricated in under an hour, with stable integration into IoT sensor systems.

By uniting molecular design, process optimization, and real-world device integration, zombie DSCs offer a compelling route to sustainable, high-performance indoor photovoltaics and self-powered electronics (see Figure 1). Envisioning a new phase of IoT, these DSCs can power small, autonomously operating sensor modules. Moreover, integrating local intelligence, such as resource-limited neural networks—allows on-device analytics and real-time energy management, boosting efficiency while relying solely on ambient light.^10,36^

**Figure 1:**
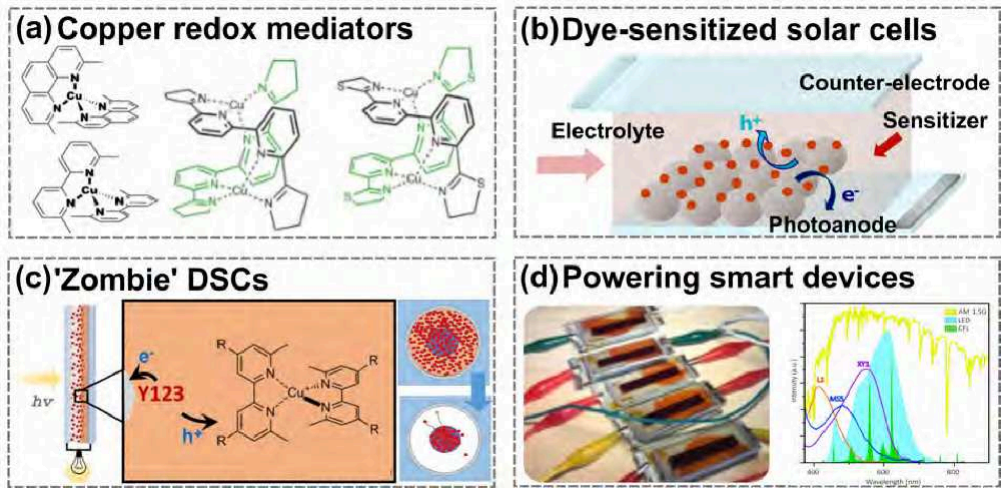
(a) Development of copper based redox shuttles. (b) Schematic representation of dye-sensitized solar cells. (c) New processes in solidification enabled via “zombiefication” of the electrolyte. (d) Efficient harvest of diffuse light by dye-sensitized solar cells enabling the powering of IoT nodes. Adapted with permission from ref 29. Copyright 2020 Springer Nature.

This Spotlight traces the materials design principles underpinning the “zombie” DSC—devices that maintain or even improve performance after the spontaneous solidification of a liquid electrolyte within the mesoporous TiO_2_ scaffold. We critically compare copper-based systems with recent breakthroughs in cobalt and iron polypyridyl complexes as well as polyiodide systems, and discuss the future outlook for scalable, sustainable indoor photovoltaics integrated with intelligent, autonomous electronics.

## Evolution of Redox Mediators: Copper Coordination Complexes

Redox mediators have been pivotal in enabling the continuous progress in DSCs, directly impacting device performance, stability, and scalability. The iodide/triiodide (I^–^/I_3_^–^) redox couple has historically set the benchmark for DSC longevity, with long-term studies demonstrating that, when paired with optimized encapsulation and ionic liquid formulations, I^–^/I_3_^–^-based devices can retain 75% of their initial performance after 12 years outdoors^13^ and experience only a 20% loss after 7000 h of ISOS-L2 testing.^12,14^ The outer-sphere reorganization energy for the rate-determining I^–^/I_3_^–^ self-exchange has been estimated to lie between 0.6 and 0.9 eV.^37^ However, direct experimental quantification is impeded by the short-lived iodine atom; we therefore omit the numerical value and instead discuss the qualitative impact of the multielectron pathway. Hence, despite the exceptional stability, the high reorganization energy and moderate redox potential of iodide limit open-circuit voltages (*V*_OC_ < 0.8 V) restrict compatibility with certain electrode materials.

To overcome these constraints, transition-metal-based redox mediators were introduced (see Figure 2), with the field-defining work of Sandra Feldt and co-workers^38^ establishing cobalt polypyridyl complexes as a new platform for higher *V*_OC_ (up to 1.0 V) and enhanced tunability. However, cobalt polypy-ridyl complexes exhibit inner-sphere reorganization energies of 0.60–0.80 eV,^39^ comparable to or slightly lower than the outer-sphere contribution in iodide systems; the overall activation barrier is nevertheless larger because the Co^III/II^ electron self-exchange is spin-forbidden and coupled to ligand-field rearrangement.^40^ The reduced Co complexes exhibit smaller diffusion coefficients (typically <10^–6^ cm^2^ s^–1^), which, along with limited solubility and reduced-state instability, contribute to performance losses, which can limit current density and overall device performance, especially in thick photoanodes.

**Figure 2:**
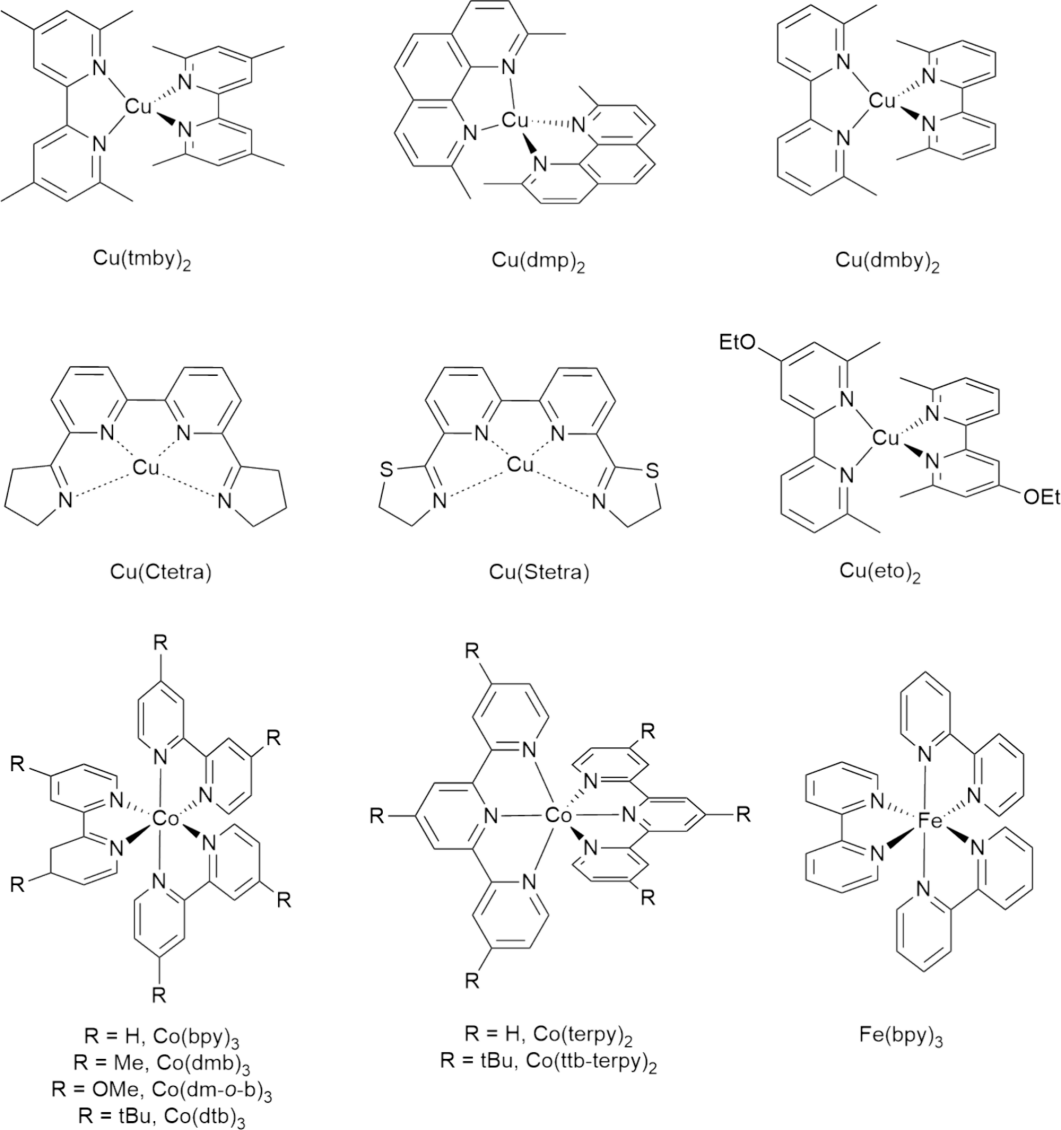
Chemical structure of copper, cobalt, and iron coordination complexes-based redox mediators implemented in DSCs. For the copper complexes shown, the relevant redox couple is generally [Cu­(ligand)_*n*_]^2+^/[Cu­(ligand)_*n*_]^+^, and the redox reaction is written as a reduction: ${\left[\mathrm{Cu}{\left(\mathrm{ligand}\right)}_{n}\right]}^{2+}+e^{-}⇌[\mathrm{Cu}(\mathrm{ligand}{)}_{n}{]}^{+}$ . Specific charge states and coordination environments depend on the particular ligand and conditions. Adapted with permission from ref 12. Copyright 2021 The Royal Society of Chemistry. And from ref 41. Copyright 2022 Elsevier.

Copper coordination complexes such as [Cu­(tmby)_2_]^2+/+^ (tmby = 4,4′,6,6′-tetramethyl-2,2′-bipyridine) and related derivatives have since emerged as especially promising redox mediators, (see Table 1) offering high redox potentials (∼0.87 V vs SHE), rapid dye regeneration with driving forces as low as 0.1 eV, and particularly low inner reorganization energies (0.16–0.40 eV).^12,17,18,20,42,43^ These low reorganization energies are directly responsible for the fast charge-transfer kinetics at the dye–electrolyte interface, delivering improved fill factors and higher photovoltages compared to cobalt-based systems.^44^ The molecular engineering of copper complexes, and in particular the introduction of sterically hindered substituents, plays a central role in stabilizing both Cu­(I) and Cu­(II) oxidation states, minimizing geometric rearrangement between pseudotetrahedral (Cu­(I)) and square-planar (Cu­(II)) geometries.^20,45^ This “rigidity” in the ligand field allows for efficient charge transfer with minimal reorganization, as confirmed by computational studies and cyclic voltammetry. For example, [Cu­(tmby)_2_]^2+/+^ exhibits a redox potential of ∼0.87 V vs SHE and a reorganization energy as low as 0.27 eV, thereby elevating the device *V*_OC_.^17,18,20,42,43^

**Table 1:** Representative Properties of Copper Redox Mediators

Complex	Ligand Type	Redox Potential (V vs SHE)	Reorganization Energy (eV)	Notes
[Cu(tmby)_2_]^2+/+^	Bipyridyl (tmby)	∼0.87	0.27–0.31^a^	High *V*_OC_; benchmark system^17^
[Cu(dmp)_2_]^2+/+^	Phenanthroline (dmp)	∼0.90	0.29^a^	Low reorganizational energy, fast dye regeneration^17^
[Cu(STetra)]^2+/+^	Tetradentate derivatives	∼0.65	0.16–0.40^b^	Exhibiting disproportionation; suppressing recombination^41^
[Cu(dmby)_2_]^2+/+^	Bipyridyl (dmby)	∼0.97	0.30^a^	Low reorganizational energy^17^
[Cu(CTetra)]^2+/+^	Tetradentate derivatives	∼0.57	0.39^b^	Exhibiting disproportionation; suppressing recombination^41^
[Cu(eto)_2_]^2+/+^	Bipyridyl (ethoxy)	∼0.86	0.32^b^	charge recombination process occuring in the Marcus inverted regime^42^

a Experimentally estimated or derived from electrochemical/kinetic studies.^17^

b Computationally derived using DFT.^41,42^

Copper diimine complexes exhibit complex solution dynamics but can be stabilized in the solid state by careful ligand design and controlled solidification. For example, methylation at the 6,6′-positions in bipyridine ligands increases steric hindrance, effectively reducing structural changes during redox cycling and improving stability.^20^ The formation of an amorphous network within the TiO_2_ scaffold, as confirmed by impedance spectroscopy, results in minimal charge transfer resistance increase during solidification.^28^ A particularly exciting development is the advent of dynamic dimer copper systems,^46^ where two Cu­(I) units form a bridged dimer that, upon oxidation, disproportionates to yield a Cu­(I) dimer and two Cu­(II) monomers. Density Functional Theory (DFT) calculations indicate inner-sphere reorganization energies as low as 0.27 eV for these processes, accelerating dye regeneration and suppressing unwanted charge recombination.

The use of copper-based mediators has enabled DSC devices to achieve record power conversion efficiencies under ambient conditions. Under simulated indoor lighting (1000 lx), DSCs employing copper complexes routinely reach PCEs of 35–38%, with *V*_OC_ exceeding 1.0 V and fill factors above 0.75.^44,47^ Recent work on asymmetric dual-species Cu­(II)/Cu­(I) electrolytes has achieved PCEs up to 35.6% under 1000 lx warm white illumination, with exceptional stability, although these remain quasi-solid rather than fully solid-state systems.^31^ The synergy between tailored dye molecules and copper complexes is essential to maximize *J*_SC_ and maintain high FF in assembled cells.

## Transition from Liquid to “Zombie” Cells

While liquid electrolytes have enabled high-performing DSCs for decades, their practical implementation is complicated by the need for robust sealing to prevent solvent leakage and ensure long-term stability, especially in flexible or large-area modules.^12–14^ Even with recent advances in ionic liquid formulations and encapsulation, gradual solvent loss or moisture ingress can degrade performance over time.

A pivotal breakthrough arrived in 2015 with the serendipitous discovery of “zombie” DSCs devices that retain high performance even after most volatile solvent has evaporated, forming an in situ quasi-solid hole-transport matrix from the copper complex electrolyte (see Figure 3).^27,28,48^ In this process, the copper complexes transition from a liquid state to a stable, partially or fully amorphous (and occasionally microcrystalline) network within the mesoporous TiO_2_ scaffold as the solvent is lost.^24,29^ This spontaneously formed solid network preserves the original device structure and often thickens conduction pathways around TiO_2_ particles, contributing to efficient electron collection and mitigating leakage risks. Recent studies underscore the critical role of interfacial engineering during the solidification process; controlled formation of the copper complex network at the TiO_2_/HTM interface reduces recombination losses and improves charge collection efficiency.^49^ The resulting “zombie” matrix is typically partially amorphous, as confirmed by X-ray diffraction, which is beneficial for hole transport: excess crystallinity can hinder hopping, while an amorphous matrix supports percolative conduction via mixed-valence pathways, with high hole mobility enabled by a controlled distribution of Cu­(I) and Cu­(II) species.^50^

**Figure 3:**
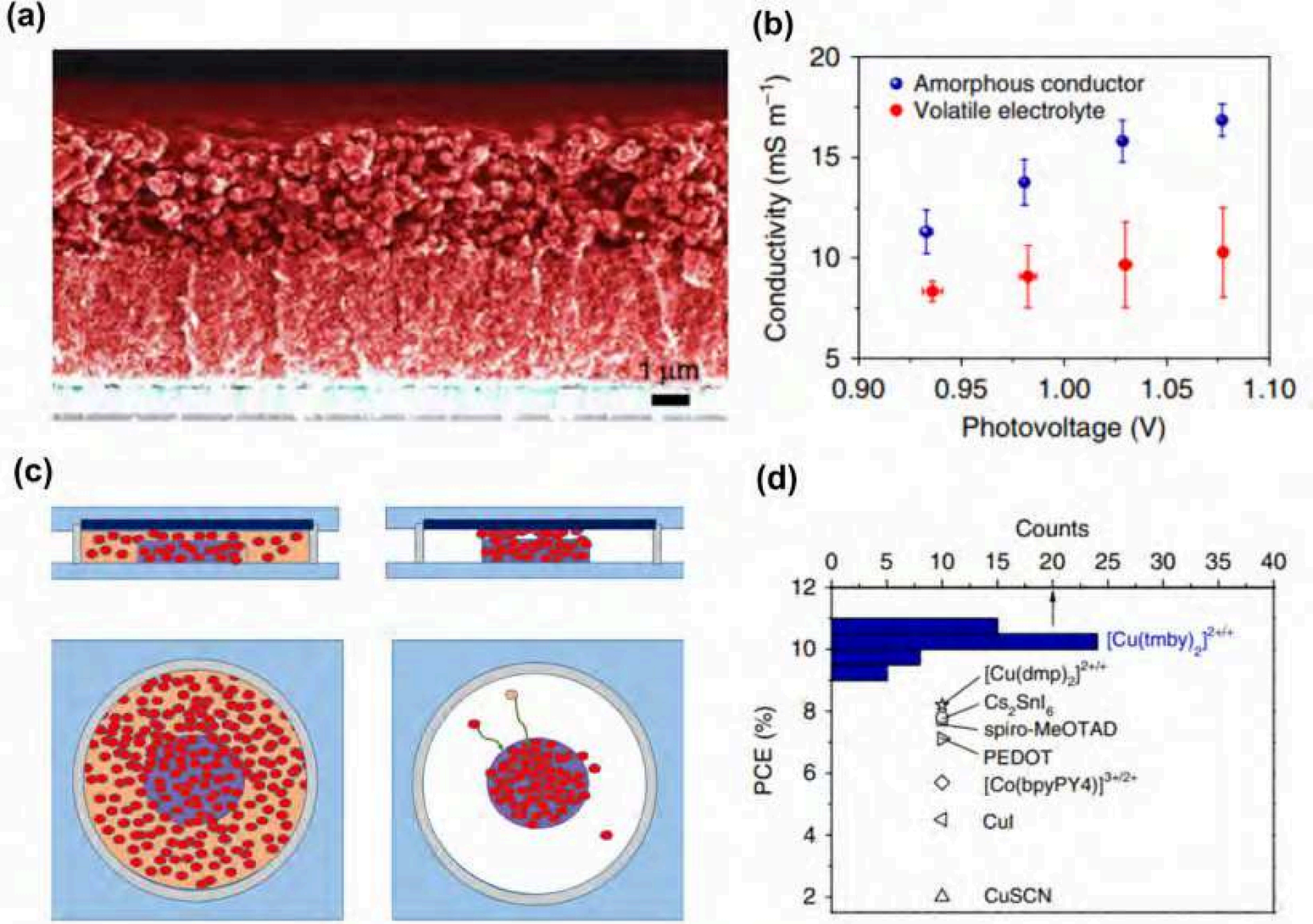
(a) Cross-sectional SEM image of a solid-state DSC without the counter electrode. Moving from the bottom to the top, layers of compact TiO_2_-coated FTO, 3.5 μm thick transparent mesoscopic TiO_2_ and 3.0 μm thick light scattering TiO_2_, and 2.0 μm thick solid Cu­(I/II) hole conductor overlayer are visible. (b) Photovoltage-dependent conductivity in the solid hole conductor and the volatile electrolyte of solar cells as obtained from the EIS analysis. (c) Schematic representations of liquid state DSC (left) and zombie device (right). (d) Histogram of PCE of solid-state HTM devices comprising a blend of Cu­(tmby)_2_^2+^ and Cu­(tmby)_2_^1+^, compared to the literature reports of commonly used HTM. Adapted with permission from ref 29. Copyright 2020 Springer Nature.

To contextualize the “zombie” approach, we compare it with other quasi-solid strategies such as polymer gel electrolytes (PGEs):

***Polymer Gel Electrolytes (PGEs):*** Immediate solidification, established manufacturing, mechanical flexibility.

*Limitations:* Lower conductivity (10^–3^–10^–4^ S cm^–1^), polymer degradation, increased interface resistance.^51^

***“Zombie” State:*** High conductivity (∼1 mS cm^–1^), direct pore filling, superior interfacial contact.

*Limitations:* Longer formation time (3–7 days under ambient conditions, reducible to <24 h with temperature/humidity control), process sensitivity.

Recent advances in polymer gel design have achieved improved stability and performance,^51^ but the “zombie” approach offers unique advantages for applications requiring high conductivity and intimate mesoporous contact. Large-area copper-based DSC modules fabricated via the “zombie” process have maintained high PCEs over time, with robust performance even after 1000 h of continuous operation under 1000 lx and temperature/humidity cycling (<5% degradation).^28,52^

Morphologically, after 3–7 days of solvent evaporation, microscopy reveals that copper complexes fill the TiO_2_ pores and form a 1–2 μm organic layer bridging the TiO_2_ to the cathode; the final thickness and continuity affect series resistance, *J*_SC_, and fill factor.^29,50^ Charge transport in the “zombie” matrix is mediated by a controlled distribution of Cu­(I) and Cu­(II) (typically 1–5% Cu­(II)), which enables percolative hole conduction rather than pure ionic diffusion.^50,53,54^ Excessive crystallization or inappropriate ligand selection can disrupt this network, reducing performance. Impedance spectroscopy confirms that efficient solidification preserves low charge transfer resistance and high fill factor.

Performance metrics illustrate minimal loss during the liquid-to-solid transition: under 1000 lx, initial PCE is 34% (*V*_OC_ = 0.91 V; *J*_SC_ = 147 μA cm^–2^; FF = 0.77); after solidification, PCE is 30% (*V*_OC_ = 0.86 V; *J*_SC_ = 137 μA cm^–2^; FF, unchanged), with less than 5% further loss over 1000 h of thermal and humidity cycling.^50^ This is attributed to the formation of an efficient mixed-valence hole transport pathway in the amorphous matrix.

The solidification period can be reduced from 3–7 days to under 24–36 h by temperature (30–50 °C) and humidity (RH, 40–60%) control, or to 12–18 h using vacuum or microwave-assisted drying. Roll-to-roll compatible processes have been demonstrated for 100 cm^2^ modules, with in-line impedance monitoring confirming uniform solidification and performance retention.^52^

## Transition from Materials to Integrated Smart Devices

The transition from materials innovation to real-world devices is most strikingly illustrated by the performance and versatility of copper-based “zombie” DSCs in powering the IoT. For autonomous sensors and microcontrollers, a stable, maintenance-free power supply in the microwatt-to-milliwatt range is essential, but traditional batteries require frequent replacement or recharging, which is costly and unsustainable at scale.^1^ “Zombie” DSCs bypass many of these limitations. Their quasi-solid matrix eliminates the need for external sealing, virtually eliminates liquid leakage, and allows for seamless integration into device casings, flexible substrates, or even textile-based electronics. Arrays of “zombie” cells in series can consistently produce 3–5 V for direct microcontroller operation, removing the necessity for voltage boosters and reducing system complexity.

Recent experiments with 25 cm^2^ arrays demonstrate robust power delivery: 88.5 μW cm^–2^ at 1000 lx and 15.6 μW cm^–2^ at 200 lx (see Figure 4), corresponding to total outputs of 2.2 mW and 0.39 mW per module, sufficient for tasks such as periodic sensor measurements, on-device computation, and wireless data transmission.^55^ These modules can charge supercapacitors or enable continuous operation, as validated in recent IoT demonstrator platforms.^56^ The quasi-solid copper matrix also improves mechanical resilience, tolerating moderate bending and thermal cycling, while dye engineering enables color tuning for product integration or camouflage in consumer electronics.^57^

**Figure 4:**
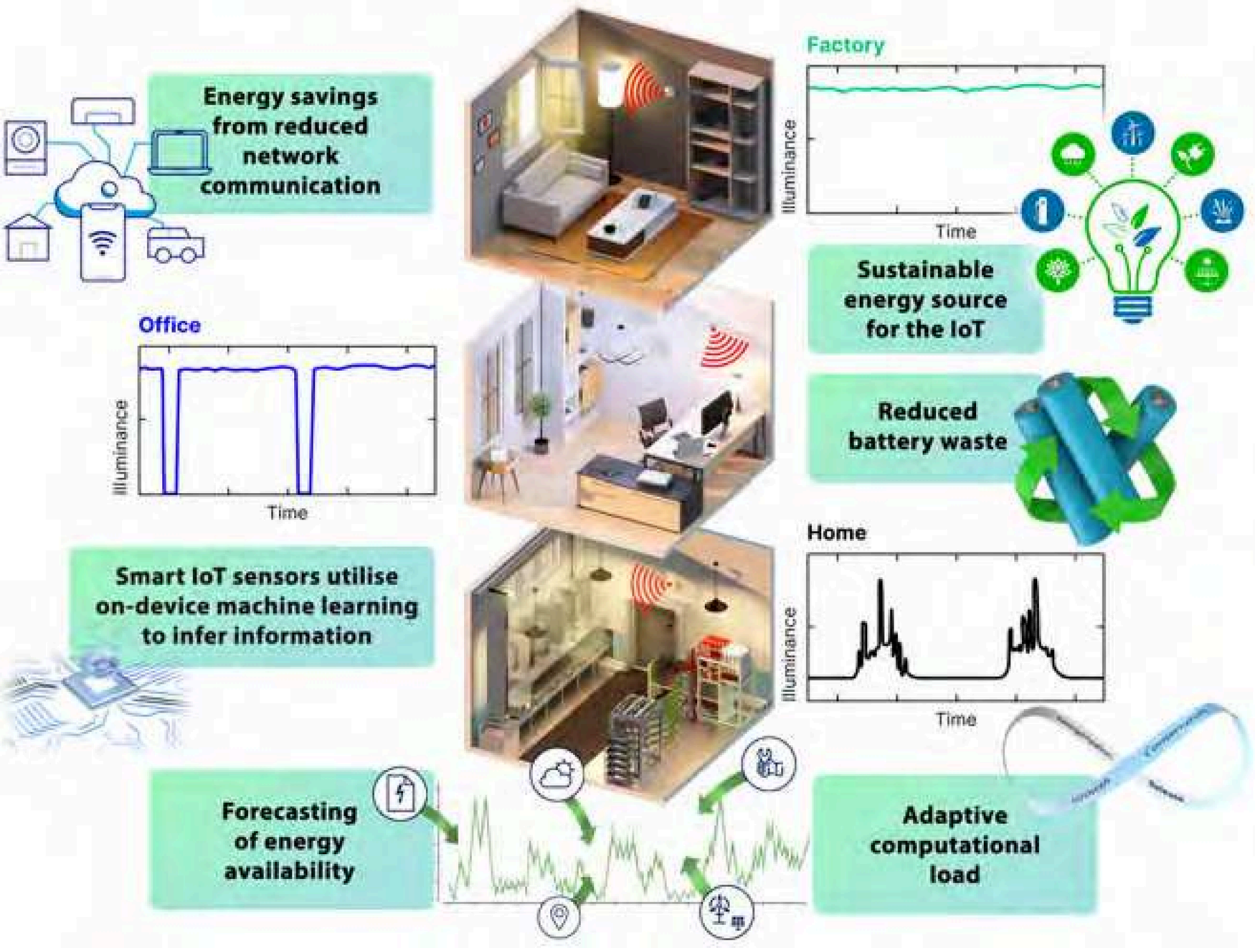
Combining high-performance indoor photovoltaics with adaptive, on-device intelligence, enabling fully self-powered IoT devices that dynamically optimize their energy usage, illustrating the synergy between materials science, AI, and sustainable sensing. Reproduced with permission from ref 30. Copyright 2025 The Royal Society of Chemistry. Credit: Ella Marushchenko, Ella Maru Studio.

The advantages of DSCs for indoor PV applications are further amplified by several fundamental factors. First, **spectral matching** plays a crucial role: indoor light sources, such as LEDs and fluorescents, emit narrow bands that overlap closely with the absorption spectra of tailored DSC dyes, enabling power conversion efficiencies up to 38% at 1000 lx, well above the 11–14% typical under AM 1.5G sunlight.^1^ Second, **reduced thermal stress** under indoor conditions mitigates many degradation pathways, as evidenced by stability studies showing <5% PCE loss after 1000 h of continuous operation, even with temperature cycling between – 10 and 50 °C or humidity fluctuations.^58^ Third, **voltage optimization** is realized in copper-based “zombie” DSCs, which maintain *V*_OC_ > 1.0 V under low-light due to reduced recombination and optimized energy alignment, a crucial feature for direct IoT powering.

The pathway to industrial scalability is now clear. Roll-to-roll manufacturing is demonstrated by companies such as Ambient Photonics and Exeger, with module areas scaling from 1 to 100 cm^2^ and beyond.^59^ Copper-based mediators are both earth-abundant and cost-effective (raw material costs as low as $8 m^–2^), supporting sustainable, large-scale deployment. Standardized, reproducible testing under commercial LED arrays (200–1000 lx) ensures robust quality control. Long-term studies show that the mixed-valence Cu­(II)/Cu­(I) charge transport network remains stable for months, with less than 5% loss in PCE over 1000 h, even under environmental cycling and partial shading.^55,58^

A paradigm shift in IoT is now underway, led by the integration of local (edge) intelligence. Zombie DSC modules directly power resource-limited microcontrollers capable of running on-device neural network inference, the emerging “tiny ML” paradigm.^24,30,60–62^ For example, a 20 cm^2^ DSC array can support continuous LSTM-based pattern recognition and BLE data transmission under typical office or factory lighting. The field prototypes show that even 4–9 cm^2^ arrays, fully encapsulated in polymer packaging, reliably power BLE or Wi-Fi sensor boards for over a year without performance loss.^30^ Real-time adaptation including maximum power point tracking, load forecasting, and lighting prediction, can be implemented to optimize both energy harvesting and information throughput. Adaptive scheduling and environmental monitoring strategies further extend application scenarios, from occupant detection and anomaly classification to haptic feedback and smart environmental control.

The “zombie” approach also enables direct, monolithic integration: modules can be roll-to-roll printed, then undergo controlled solvent removal to form the solid-state matrix, all without complex fluid injection or postassembly sealing. On-chip intelligence can dynamically manage partial shading, lighting variation, and even device aging, closing the loop between energy supply and computational demand.^63^ Early pilots in building- or appliance-integrated modules are underway, with DSC clusters reliably supporting occupant detection and environmental feedback in real-world settings.

## Future Outlook, Scalability, and Conclusions

Despite recent advances, large-area fabrication and industrial implementation of “zombie” DSCs still face several important challenges. Achieving uniform infiltration and solidification of copper complexes throughout thick (>10 μm) TiO_2_ scaffolds is particularly critical for scaling up. Pilot-scale demonstrations have shown that modules up to 100 cm^2^ can be fabricated, maintaining power conversion efficiencies (PCE) of 30–35% at 1000 lx, with less than 5% loss after 1000 h of indoor operation and environmental cycling (−10 to +50 °C, 30–90% RH).^28,52,55^ The solidification period, previously a barrier for industrial scale-up, has now been reduced from 3 to 7 days to under 24–36 h by employing temperature/humidity control or 12–18 h with vacuum-assisted drying.^29,34^ Roll-to-roll compatible processes and in-line impedance monitoring are being developed to ensure high throughput and quality control for mass production.

Materials innovation is also accelerating. Copper-based coordination polymers (CPs), especially iodide-bridged systems, have been shown to combine high conductivities (∼1 mS cm^–1^) and room-temperature hole mobilities (5.8 × 10^–4^ cm^2^ V^–1^ s^–1^) with selective band-like transport.^50,64^ These CPs can be processed from solution with minimal additives, reducing variability and potential for phase segregation. Their extended copper-halide motifs facilitate efficient hole conduction even in semiamorphous films, and their low overpotentials for dye regeneration further contribute to device stability and reproducibility. Optimizing polymer chain orientation and the polymer–electrode interface remains an active area of research, with the goal of achieving high-performance, cost-effective solid-state DSCs for both indoor and outdoor applications.

The mechanistic understanding of DSC operation under low-light is improving, with detailed balance analysis predicting theoretical PCE limits of 52–58% for single-junction DSCs under indoor spectra.^4,64^ State-of-the-art copper-based “zombie” DSCs have achieved PCEs of 34–38% at 1000 lx, fill factors exceeding 0.75, and open-circuit voltages above 1.0 V.^55,65^ Monochromatic external quantum efficiencies of up to 91% have been reported at 530 nm. Notably, large-area rapid-zombie polyiodide DSCs can be fabricated in under an hour and show 12% increases in PCE over 12 months of dark storage, while liquid cells exhibit sharp declines.^34^ These results highlight the importance of controlling crystallinity, oxidation state distribution, and the interplay of ligand structure with charge carrier concentration for sustained high performance.

Recent developments in embedded artificial intelligence (TinyML) are enabling local inference and adaptive workload management in self-powered IoT nodes, directly powered by “zombie” DSC modules as small as 4–9 cm^2^.^30,66,67^ These sensor nodes have been shown to operate for over a year under variable indoor lighting, with experimental power outputs (e.g., 88.5 μW cm^–2^ at 1000 lx and 15.6 μW cm^–2^ at 200 lx) sufficient for wireless communication and local analytics. The environmental benefit is clear: no single-use batteries, no rare or toxic elements, and a path to long-term, maintenance-free operation. The practical integration of DSCs with roll-to-roll manufacturing, and their robust performance under real-world conditions, is now being validated by companies such as Ambient Photonics and Exeger.^59^

The evolution and partial crystallization of the copper matrix can either enhance or hinder conduction, depending on the ligand environment and process conditions. Careful optimization is needed to balance the doping fraction, avoid overoxidation, and maintain high hole mobility. Scaling uniform performance to flexible substrates and large areas (50–500 cm^2^) without introducing defects or nonuniformities is a key challenge for commercial adoption. Ongoing studies into ligand structure, charge-carrier concentration, and the interplay with coordination polymer frameworks hold promise for the next generation of high-efficiency, robust DSCs.^55,64,65^

In summary, copper coordination complexes have propelled DSCs from moderate-voltage, leakage-prone liquid systems to robust, high-voltage quasi-solid architectures ready for sustainable indoor and ambient photovoltaics. By exploiting low reorganization energies and controlled oxidation state distributions, today’s DSC modules deliver 10–14% PCE under simulated sunlight and 34–38% under 200–1000 lx indoor lighting. The “zombie” DSC concept, where liquid electrolyte transforms into a stable, high-conductivity solid, addresses persistent challenges of leakage, mass transport, and manufacturing complexity. As advances in coordination chemistry, device engineering, and embedded AI converge, copper-based DSCs are poised to expand the frontier of sustainable photovoltaics and enable a new generation of intelligent, autonomous IoT systems.

Looking forward, as copper-driven dye-sensitized solar cells move seamlessly from laboratory innovation to real-world deployment, they are not only redefining the boundaries of ambient photovoltaics but also laying the foundation for a future where self-powered, adaptive devices become an invisible, enduring fabric of everyday life.

## References

[1] Pecunia V., Silva S., Phillips J., Artegiani E., Romeo A., Shim H., Park J., Kim J., Yun J., Welch G., Larson B., Creran M., Laventure A., Sasitharan K., Flores-Diaz N., Freitag M., Xu J., Brown T., Li B., Wang Y., Li Z., Hou B., Hamadani B., Defay E., Kovacova V., Glinsek S., Kar-Narayan S., Bai Y., Kim D., Cho Y., Žukauskaitė A., Barth S., Fan F., Wu W., Costa P., del Campo J., Lanceros-Mendez S., Khanbareh H., Wang Z., Pu X., Pan C., Zhang R., Xu J., Zhao X., Zhou Y., Chen G., Tat T., Ock I., Chen J., Graham S., Yu J., Huang L.-Z., Li D.-D., Ma M.-G., Luo J., Jiang F., Lee P., Dudem B., Vivekananthan V., Kanatzidis M., Xie H., Shi X.-L., Chen Z.-G., Riss A., Parzer M., Garmroudi F., Bauer E., Zavanelli D., Brod M., Malki M., Snyder G., Kovnir K., Kauzlarich S., Uher C., Lan J., Lin Y.-H., Fonseca L., Morata A., Martin-Gonzalez M., Pennelli G., Berthebaud D., Mori T., Quinn R., Bos J., Candolfi C., Gougeon P., Gall P., Lenoir B., Venkateshvaran D., Kaestner B., Zhao Y., Zhang G., Nonoguchi Y., Schroeder B., Bilotti E., Menon A., Urban J., Fenwick O., Asker C., Talin A., Anthopoulos T., Losi T., Viola F., Caironi M., Georgiadou D., Ding L., Peng L.-M., Wang Z., Wei M.-D., Negra R., Lemme M., Wagih M., Beeby S., Ibn-Mohammed T., Mustapha K., Joshi A. (2023). Roadmap on
energy harvesting materials. JPhys. Materials.

[2] Hittinger E., Jaramillo P. (2019). Internet of
Things: Energy boon or bane?. Science.

[3] Anctil A., Beattie M., Case C., Chaudhary A., Chrysler B., Debije M., Essig S., Ferry D., Ferry V., Freitag M., Gould I., Hinzer K., Hoppe H., Inganas O., Krishnan
Jagadamma L., Hun Jee M., Kostuk R., Kirk D., Kube S., Lim M., Luther J., Mansfield L., McGehee M., Nguyen Minh D., Nain P., Reese M., Reinders A., Samuel I., Van Sark W., Savin H., Sellers I., Shaheen S., Tang Z., Toor F., Vahanissi V., Wassweiler E., Warren E., Whiteside V., Woo H., Xiong G., Zhu X. (2023). Status report on emerging photovoltaics. J. Photonics Energy.

[4] Li B., Hou B., Amaratunga G. A. J. (2021). Indoor photovoltaics, The Next Big
Trend in solution-processed solar cells. InfoMat.

[5] Wang W., Cui Y., Zhang T., Bi P., Wang J., Yang S., Wang J., Zhang S., Hou J. (2023). High-performance organic
photovoltaic cells under indoor lighting enabled by suppressing energetic
disorders. Joule.

[6] Sasitharan K., Kilbride R. C., Spooner E. L., Clark J., Iraqi A., Lidzey D. G., Foster J. A. (2022). Metal-Organic
Framework Nanosheets
as Templates to Enhance Performance in Semi-Crystalline Organic Photovoltaic
Cells. Adv. Sci..

[7] Sasitharan K., Frisch J., Kuliček J., Iraqi A., Lidzey D. G., Bär M., Rezek B., Foster J. A. (2024). Tuning the morphology
and energy levels in organic solar cells with metal-organic framework
nanosheets. Sci. Rep..

[8] Li M., Zhao C., Wang Z., Zhang C., Lee H. K. H., Pockett A., Barbé J., Tsoi W. C., Yang Y., Carnie M. J., Gao X., Yang W., Durrant J. R., Liao L., Jain S. M. (2018). Interface
Modification by Ionic Liquid:
A Promising Candiyear for Indoor Light Harvesting and Stability Improvement
of Planar Perovskite Solar Cells. Adv. Energy
Mater..

[9] Ma Q., Wang Y., Liu L., Yang P., He W., Zhang X., Zheng J., Ma M., Wan M., Yang Y., Zhang C., Mahmoudi T., Wu S., Liu C., Hahn Y.-B., Mai Y. (2024). One-step dual-additive
passivated
wide-bandgap perovskites to realize 44.72%-efficient indoor photovoltaics. Energy Environ. Sci..

[10] Michaels H., Benesperi I., Freitag M. (2021). Challenges and prospects of ambient
hybrid solar cell applications. Chem. Sci..

[11] O’Regan B., Gratzel M. (1991). A low-cost, high-efficiency
solar cell based on dye-sensitized
colloidal TiO2 films. Nature.

[12] Muñoz-García A., Benesperi I., Boschloo G., Concepcion J., Delcamp J., Gibson E., Meyer G., Pavone M., Pettersson H., Hagfeldt A., Freitag M. (2021). Dye-sensitized solar
cells strike back. Chem. Soc. Rev..

[13] Kato N., Tanaka H., Takeda Y., Higuchi K., Nakajima J. (2023). Demonstration
of 12 Years Outdoor Working of Highly Durable Dye-Sensitized Solar
Cell Modules Employing Hydrophobic Surface Passivation and Suppression
of Biased Distribution of Iodine Ions. ACS Sustainable
Chem. Eng..

[14] Godfroy M., Liotier J., Mwalukuku V. M., Joly D., Huaulmé Q., Cabau L., Aumaitre C., Kervella Y., Narbey S., Oswald F., Palomares E., Gonzalez Flores C. A. G., Oskam G., Demadrille R. (2021). Benzothiadiazole-based photosensitizers
for efficient and stable dye-sensitized solar cells and 8.7% efficiency
semi-transparent mini-modules. Sustainable Energy
Fuels.

[15] Hattori S., Wada Y., Yanagida S., Fukuzumi S. (2005). Blue copper model complexes
with distorted tetragonal geometry acting as effective electron-transfer
mediators in dye-sensitized solar cells. J.
Am. Chem. Soc..

[16] Colombo A., Dragonetti C., Magni M., Roberto D., Demartin F., Caramori S., Bignozzi C. A. (2014). Efficient Copper Mediators Based
on Bulky Asymmetric Phenanthrolines for DSSCs. ACS Appl. Mater. Interfaces.

[17] Saygili Y., Söderberg M., Pellet N., Giordano F., Cao Y., Munoz-García A., Zakeeruddin S., Vlachopoulos N., Pavone M., Boschloo G., Kavan L., Moser J.-E., Grätzel M., Hagfeldt A., Freitag M. (2016). Copper Bipyridyl Redox
Mediators for Dye-Sensitized Solar Cells with High Photovoltage. J. Am. Chem. Soc..

[18] Michaels H., Benesperi I., Edvinsson T., Muñoz-Garcia A. B., Pavone M., Boschloo G., Freitag M. (2018). Copper Complexes with
Tetradentate Ligands for Enhanced Charge Transport in Dye-Sensitized
Solar Cells. Inorganics.

[19] Saygili Y., Turren-Cruz S.-H., Olthof S., Saes B., Pehlivan I., Saliba M., Meerholz K., Edvinsson T., Zakeeruddin S., Grätzel M., Correa-Baena J.-P., Hagfeldt A., Freitag M., Tress W. (2018). Planar Perovskite Solar
Cells with High Open-Circuit Voltage Containing a Supramolecular Iron
Complex as Hole Transport Material Dopant. ChemPhysChem.

[20] Freitag M., Giordano F., Yang W., Pazoki M., Hao Y., Zietz B., Grätzel M., Hagfeldt A., Boschloo G. (2016). Copper phenanthroline
as a fast and high-performance redox mediator for dye-sensitized solar
cells. J. Phys. Chem. C.

[21] Benesperi I., Singh R., Freitag M. (2020). Copper coordination
complexes for
energy-relevant applications. Energies.

[22] Ren Y., Zhang D., Suo J., Cao Y., Eickemeyer F. T., Vlachopoulos N., Zakeeruddin S. M., Hagfeldt A., Grätzel M. (2023). Hydroxamic
acid pre-adsorption raises the efficiency of cosensitized solar cells. Nature.

[23] Ren Y., Li Y., Chen S., Liu J., Zhang J., Wang P. (2016). Improving
the performance of dye-sensitized solar cells with electron-donor
and electron-acceptor characteristic of planar electronic skeletons. Energy Environ. Sci..

[24] Michaels H., Rinderle M., Freitag R., Benesperi I., Edvinsson T., Socher R., Gagliardi A., Freitag M. (2020). Dye-sensitized solar cells under ambient light powering
machine learning: Towards autonomous smart sensors for the internet
of things. Chem. Sci..

[25] Freitag M., Teuscher J., Saygili Y., Zhang X., Giordano F., Liska P., Hua J., Zakeeruddin S., Moser J.-E., Grätzel M., Hagfeldt A. (2017). Dye-sensitized solar
cells for efficient power generation under ambient lighting. Nat. Photonics.

[26] Devdass A., Watson J., Firestone E., Hamann T. W., Delcamp J. H., Jurss J. W. (2022). An Efficient Copper-Based Redox Shuttle Bearing a Hexadentate
Polypyridyl Ligand for DSCs under Low-Light Conditions. ACS Appl. Energy Mater..

[27] Freitag M., Daniel Q., Pazoki M., Sveinbjörnsson K., Zhang J., Sun L., Hagfeldt A., Boschloo G. (2015). High-efficiency
dye-sensitized solar cells with molecular copper phenanthroline as
solid hole conductor. Energy Environ. Sci..

[28] Saygili Y., Stojanovic M., Kim H.-S., Teuscher J., Scopelliti R., Freitag M., Zakeeruddin S. M., Moser J.-E., Gratzel M., Hagfeldt A. (2020). Liquid State and Zombie Dye Sensitized Solar Cells
with Copper Bipyridine Complexes Functionalized with Alkoxy Groups. J. Phys. Chem. C.

[29] Cao Y., Saygili Y., Ummadisingu A., Teuscher J., Luo J., Pellet N., Giordano F., Zakeeruddin S., Moser J.-E., Freitag M., Hagfeldt A., Grätzel M. (2017). 11% efficiency
solid-state dye-sensitized solar cells with copper­(II/I) hole transport
materials. Nat. Commun..

[30] Michaels H., Rinderle M., Benesperi I., Freitag R., Gagliardi A., Freitag M. (2023). Emerging indoor photovoltaics for self-powered and
self-aware IoT towards sustainable energy management. Chem. Sci..

[31] Meethal S. M., Pradhan S. C., Velore J., Varughese S., Pillai R. S., Sauvage F., Hagfeldt A., Soman S. (2024). Asymmetric
dual species copper­(ii/i) electrolyte dye-sensitized solar cells with
35.6% efficiency under indoor light. J. Mater.
Chem. A.

[32] Kashif M.
K., Milhuisen R. A., Nippe M., Hellerstedt J., Zee D. Z., Duffy N. W., Halstead B., De Angelis F., Fantacci S., Fuhrer M. S., Chang C. J., Cheng Y.-B., Long J. R., Spiccia L., Bach U. (2016). Cobalt Polypyridyl
Complexes as Transparent Solution-Processable Solid-State Charge Transport
Materials. Adv. Energy Mater..

[33] Kashif M. K., Benesperi I., Milhuisen R. A., Meyer S., Hellerstedt J., Zee D. Z., Duffy N. W., Halstead B., Fuhrer M. S., Cashion J., Cheng Y.-B., Spiccia L., Simonov A. N., Bach U. (2017). Polypyridyl Iron Complex as a Hole-Transporting Material for Formamidinium
Lead Bromide Perovskite Solar Cells. ACS Energy
Lett..

[34] Sutton M., Lei B., Michaels H., Freitag M., Robertson N. (2022). Rapid and
Facile Fabrication of Polyiodide Solid-State Dye-Sensitized Solar
Cells Using Ambient Air Drying. ACS Appl. Mater.
Interfaces.

[35] Spinelli G., Morritt G. H., Pavone M., Probert M. R., Waddell P. G., Edvinsson T., Muñoz-García A. B., Freitag M. (2023). Conductivity
in Thin Films of Transition Metal Coordination Complexes. ACS Appl. Energy Mater..

[36] Mathews I., Kantareddy S. N., Buonassisi T., Peters I. M. (2019). Technology and Market
Perspective for Indoor Photovoltaic Cells. Joule.

[37] Wang X., Stanbury D. M. (2006). Oxidation of Iodide
by a Series of Fe­(III) Complexes
in Acetonitrile. Inorg. Chem..

[38] Feldt S. M., Gibson E. A., Gabrielsson E., Sun L., Boschloo G., Hagfeldt A. (2010). Design of Organic Dyes and Cobalt
Polypyridine Redox
Mediators for High-Efficiency Dye-Sensitized Solar Cells. J. Am. Chem. Soc..

[39] Ørnsø K. B., Jónsson E., Jacobsen K. W., Thygesen K. S. (2015). Importance of the
Reorganization Energy Barrier in Computational Design of Porphyrin-Based
Solar Cells with Cobalt-Based Redox Mediators. J. Phys. Chem. C.

[40] Feldt S. M., Wang G., Boschloo G., Hagfeldt A. (2011). Effects of Driving
Forces for Recombination and Regeneration on the Photovoltaic Performance
of Dye-Sensitized Solar Cells using Cobalt Polypyridine Redox Couples. J. Phys. Chem. C.

[41] Benesperi I., Michaels H., Edvinsson T., Pavone M., Probert M., Waddell P., Muñoz-García A., Freitag M. (2022). Dynamic dimer
copper coordination redox shuttles. Chem..

[42] Saygili Y., Stojanovic M., Michaels H., Tiepelt J., Teuscher J., Massaro A., Pavone M., Giordano F., Zakeeruddin S., Boschloo G., Moser J.-E., Grätzel M., Muñoz-García A., Hagfeldt A., Freitag M. (2018). Effect of
Coordination Sphere Geometry of Copper Redox Mediators on Regeneration
and Recombination Behavior in Dye-Sensitized Solar Cell Applications. ACS Applied Energy Materials.

[43] Saygili Y., Stojanovic M., Flores-Díaz N., Zakeeruddin S. M., Vlachopoulos N., Grätzel M., Hagfeldt A. (2019). Metal Coordination
Complexes as Redox Mediators in Regenerative Dye-Sensitized Solar
Cells. Inorganics.

[44] Wang Y., Hamann T. W. (2018). Improved Performance Induced by in
situ Ligand Exchange
Reactions of Copper Bipyridyl Redox Couples in Dye-Sensitized Solar
Cells. Chem. Commun..

[45] Rodrigues R. R., Lee J. M., Taylor N. S., Cheema H., Chen L., Fortenberry R. C., Delcamp J. H., Jurss J. W. (2020). Copper-based redox
shuttles supported by preorganized tetradentate ligands for dye-sensitized
solar cells. Dalton Trans..

[46] Benesperi I., Michaels H., Edvinsson T., Pavone M., Probert M. R., Waddell P., Muñoz-García A. B., Freitag M. (2022). Dynamic dimer
copper coordination redox shuttles. Chem..

[47] Conradie J. (2022). Polypyridyl
copper complexes as dye sensitizer and redox mediator for dye-sensitized
solar cells. Electrochem. Commun..

[48] Benesperi I., Michaels H., Freitag M. (2018). The researcher’s
guide to
solid-state dye-sensitized solar cells. J. Mater.
Chem. C.

[49] Hu M., Shen J., Yu Z., Liao R.-Z., Gurzadyan G. G., Yang X., Hagfeldt A., Wang M., Sun L. (2018). Efficient
and Stable Dye-Sensitized Solar Cells Based on a Tetradentate Copper­(II/I)
Redox Mediator. ACS Appl. Mater. Interfaces.

[50] Michaels H., Golomb M., Kim B., Edvinsson T., Cucinotta F., Waddell P., Probert M., Konezny S., Boschloo G., Walsh A., Freitag M. (2022). Copper coordination
polymers with selective hole conductivity. J.
Mater. Chem. A.

[51] Li W., Wang Y., Liu R., Chen W., Zhang H., Zhang Z. (2023). Gel Polymer-Based Composite Solid-State Electrolyte for Long-Cycle-Life
Rechargeable Zinc-Air Batteries. ACS Sustainable
Chem. Eng..

[52] Stergiopoulos T., Falaras P. (2012). Minimizing Energy Losses in Dye-Sensitized Solar Cells
Using Coordination Compounds as Alternative Redox Mediators Coupled
with Appropriate Organic Dyes. Adv. Energy Mater..

[53] Magni M., Giannuzzi R., Colombo A., Cipolla M. P., Dragonetti C., Caramori S., Carli S., Grisorio R., Suranna G. P., Bignozzi C. A., Roberto D., Manca M. (2016). Tetracoordinated Bis-phenanthroline
Copper-Complex Couple as Efficient Redox Mediators for Dye Solar Cells. Inorg. Chem..

[54] Sasitharan K., Freitag M. (2023). Nanostructured Coordination Polymers
for Solid State
Dye-Sensitized Solar Cells. ECS Meeting Abstracts.

[55] Michaels H., Rinderle M., Benesperi I., Freitag R., Gagliardi A., Freitag M. (2023). Emerging indoor photovoltaics
for self-powered and
self-aware IoT towards sustainable energy management. Chem. Sci..

[56] Flores-Diaz N., De Rossi F., Das A., Deepa M., Brunetti F., Freitag M. (2023). Progress of Photocapacitors. Chem. Rev..

[57] Sasitharan K., Mora Abarca A. J., Cucinotta F., Pineda L. W., Soto
Tellini V. H., Freitag M. (2025). Bile acid derivatives as novel co-adsorbents
for enhanced performance of blue dye-sensitized solar cells. Commun. Chem..

[58] Michaels H., Benesperi I., Freitag M. (2021). Challenges and prospects of ambient
hybrid solar cell applications. Chem. Sci..

[59] Patel P. (2024). Indoor Solar
Cells: Coming Soon to a Gadget Near You. Chem.
Eng. News.

[60] Hinton G. E., Salakhutdinov R. R. (2006). Reducing the dimensionality of data with neural networks. Science.

[61] Lai, L. ; Suda, N. Enabling Deep Learning at the IoT Edge. Proceedings of the 2018 IEEE/ACM International Conference on Computer-Aided Design (ICCAD ’18), San Diego, CA, USA; Association for Computing Machinery (ACM), 2018; 1–6. 10.1145/3240765.3243473.

[62] Abadi, M. ; Barham, P. ; Chen, J. ; Chen, Z. ; Davis, A. ; Dean, J. ; Devin, M. ; Ghemawat, S. ; Irving, G. ; Isard, M. ; Kudlur, M. ; Levenberg, J. ; Monga, R. ; Moore, S. ; Murray, D. G. ; Steiner, B. ; Tucker, P. ; Vasudevan, V. ; Warden, P. ; Wicke, M. ; Yu, Y. ; Zheng, X. TensorFlow: A System for Large-Scale Machine Learning. 12th USENIX Symposium on Operating Systems Design and Implementation (OSDI 16); USENIX Association, 2016; 265–283.

[63] Gupta N., Vaisla K. S., Kumar R. (2021). Design of a Structured
Hypercube
Network Chip Topology Model for Energy Efficiency in Wireless Sensor
Network Using. SN Comput. Sci..

[64] Spinelli G., Freitag M., Benesperi I. (2023). What is necessary
to fill the technological
gap to design sustainable dye-sensitized solar cells? Sustain. Energy Fuels.

[65] Müller D., Jiang E., Rivas-Lazaro P., Baretzky C., Loukeris G., Bogati S., Paetel S., Irvine S. J. C., Oklobia O., Jones S., Lamb D., Richter A., Siefer G., Lackner D., Helmers H., Teixeira C., Forgács D., Freitag M., Bradford D., Shen Z., Zimmermann B., Würfel U. (2023). Indoor Photovoltaics for the Internet-of-Things - A
Comparison of State-of-the-Art Devices from Different Photovoltaic
Technologies. ACS Appl. Energy Mater..

[66] Liu H., Wei Z., Zhang H., Li B., Zhao C. (2022). Tiny Machine Learning
(Tiny-ML) for Efficient Channel Estimation and Signal Detection. IEEE Trans. Veh. Technol..

[67] Sabovic A., Aernouts M., Subotic D., Fontaine J., De Poorter E., Famaey J. (2023). Towards energy-aware
tinyML on battery-less IoT devices. Internet
of Things.

